# Stable germline transgenesis using the *Minos* Tc1/*mariner* element in the sea urchin *Lytechinus pictus*

**DOI:** 10.1242/dev.202991

**Published:** 2024-08-19

**Authors:** Elliot W. Jackson, Emilio Romero, Svenja Kling, Yoon Lee, Evan Tjeerdema, Amro Hamdoun

**Affiliations:** Center for Marine Biotechnology and Biomedicine, Scripps Institution of Oceanography, University of California San Diego, La Jolla, CA 92037, USA

**Keywords:** Transgenics, Sea urchins, Transposons, Echinoderm, Minos, Pictus

## Abstract

Stable transgenesis is a transformative tool in model organism biology. Although the sea urchin is one of the oldest animal models in cell and developmental biology, studies in this animal have largely relied on transient manipulation of wild animals, without a strategy for stable transgenesis. Here, we build on recent progress to develop a more genetically tractable sea urchin species, *Lytechinus pictus*, and establish a robust transgene integration method. Three commonly used transposons (*Minos*, *Tol2* and *piggyBac*) were tested for non-autonomous transposition, using plasmids containing a polyubiquitin promoter upstream of a H2B-mCerulean nuclear marker. *Minos* was the only transposable element that resulted in significant expression beyond metamorphosis. F_0_ animals were raised to sexual maturity, and spawned to determine germline integration and transgene inheritance frequency, and to characterize expression patterns of the transgene in F_1_ progeny. The results demonstrate transgene transmission through the germline, the first example of a germline transgenic sea urchin and, indeed, of any echinoderm. This milestone paves the way for the generation of diverse transgenic resources that will dramatically enhance the utility, reproducibility and efficiency of sea urchin research.

## Teaser

**Summary:** The creation of the first germline transgenic sea urchin is expected to significantly improve the utility, reproducibility, and efficiency of sea urchin research.

## INTRODUCTION

Sea urchins are a classical animal model in cell and developmental biology. Sea urchins sit at an informative phylogenetic position as basal deuterostomes, and offer specific biological advantages as animal models, including extreme fecundity, optical transparency and clockwork synchrony of development. Research in these animals has informed mechanisms of fertilization, cell division and early development. Examples include the discovery of cyclins (Evans et al., 1983), which ultimately led to currently approved drugs targeting metastatic cancers (Petroni et al., 2020), and the first descriptions of gene regulatory networks (Davidson et al., 2002; Yuh et al., 1998), which have become a foundational framework for understanding development (Davidson and Levine, 2008; Levine and Davidson, 2005; Martik et al., 2016). Sea urchins remain a favorite for study across diverse fields, including gene regulation of development (Mcclay, 2011; McClay et al., 2020; Skvortsova et al., 2022), innate immunity (Barela Hudgell et al., 2022; Buckley and Rast, 2015; Buckley and Yoder, 2022; Fleming et al., 2021; Perillo et al., 2020; Schuh et al., 2019), biomineralization (DiCorato et al., 2018; Hijaze et al., 2024; Hu and Stumpp, 2023; Piacentino et al., 2016; Sampilo and Song, 2024), nervous system development (Angerer et al., 2011; Hinman and Burke, 2018; McClay, 2022), reproduction (Chassé et al., 2018; Wessel et al., 2014, 2021) and developmental physiology (Lee et al., 2023; Li et al., 2023).

As with several other model organisms, there are multiple species of sea urchins used by the sea urchin community. Most were chosen long ago, based on availability of the wild animals, and many have long generation times that make them poorly suited for stable genetics. The advent of CRISPR in sea urchins (Lin and Su, 2016; Oulhen and Wessel, 2016) sparked interest in development of a genetically enabled sea urchin. Two species that showed promise were *Lytechinus pictus*, which is native to the California coast, and *Temnopleurus reevesii*, which is native to Japan (Hinegardner, 1969; Yaguchi and Yaguchi, 2022). Both species can be cultured from egg-to-egg in the lab, grow at room temperature (∼20°C) and have modest adult sizes suitable for culturing in standard recirculating systems (Nesbit et al., 2019; Yaguchi and Yaguchi, 2022). As such, these are the only two species worldwide that have been used to create homozygous mutant knockout lines using CRISPR/Cas9 (Vyas et al., 2022; Yaguchi et al., 2020). However, until this study, stable transgenesis had not been demonstrated in any echinoderm, and removal of this bottleneck remained a high priority.

The current methods for expressing foreign genes in sea urchins have been through microinjection of bacterial artificial chromosomes (BACs) or synthetic mRNAs (Buckley et al., 2018; von Dassow et al., 2019). However, one major limitation of BACs and synthetic mRNAs is the transient nature of the experiment. Each experiment typically uses embryos from a new male/female wild-type mate pair and is terminated well before an F_1_ generation is reached. Stable transgenesis, defined as germline modifications in a F_1_ population and beyond, can overcome this limitation through the use of endonucleases or a transposon to incorporate foreign DNA into the genome.

In this study, we report the generation of the first germline transgenic sea urchin using transposon-mediated integration in *L. pictus*. To achieve this, a polyubiquitin promoter driving expression of a fluorescent nuclear marker was used to test three DNA transposons for non-autonomous integration. We identified *Minos* Tc1/*mariner* as the only transposable element that resulted in significantly higher integration rates when compared with controls. We then leveraged this finding to compare the transgene expression patterns across larval development to determine differences between transient expression of the plasmid and stable transgene expression from the genome. Finally, we examined transgene expression patterns between F_0_ and F_1_ progeny. This study paves the way for a new era in the generation of diverse sea urchin lines useful in cell fate analysis, live imaging, conditional gene expression and combinatorial mutagenesis.

## RESULTS

**Fig. 1.**
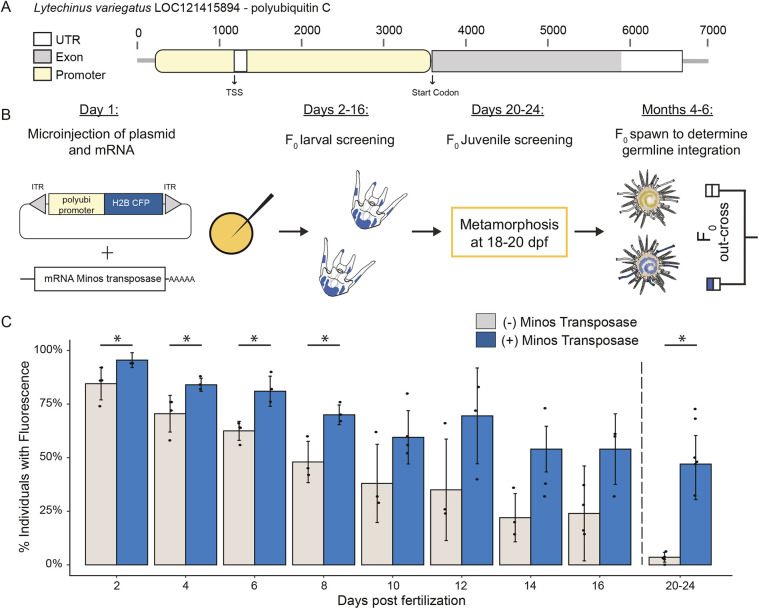
**Integrated transgenes are expressed robustly at the late larval and juvenile stages.** Transient transgene expression from the reporter construct alone was distinct from genomic transgene expression from bona fide integration at the late larval and juvenile phases. (A) Schematic of the polyubiquitin promoter from *L. variegatus* used to drive transgene expression. (B) Experimental design for creating F_0_ sea urchins with somatic integrations that could subsequently be selected for grow out. (C) Transgene expression patterns with and without the *Minos* transposase over early development. Each data point represents the proportion of offspring with fluorescence from a unique mate pair. In total, 200 larvae were screened for each timepoint from three or four different mate pairs. Data are mean±s.d. Black dashed line represents the transition from larval to juvenile through metamorphosis. *P<0.05 (Welch's two-sample *t*-test).

To design a suitable transgenesis strategy for sea urchins, we first needed to discover a promoter to drive strong ubiquitous transgene expression for efficient screening purposes. Using previous RNA-seq data, we selected putative promoters that are strongly expressed in both larval and juvenile phases to readily select juveniles with somatic integrations. Of the five promoters tested, the promoter of an *L. variegatus* polyubiquitin-C gene (LOC121415894), cloned upstream of a cyan fluorescent protein (CFP) mCerulean (LvPolyUb::H2B-CFP), was the only one that was expressed reliably in our hands. This promoter is 3439 bp and spans approximately −1000 nucleotides upstream of the transcriptional start site to the start codon in the *L. variegatus* genome (Fig. 1).

Using this construct with the *Minos* transposase, which had previously been demonstrated to have excision activity in sea urchin embryos (Sasakura et al., 2010) and had been used to achieve effective transgenesis in a related invertebrate, *Ciona intestinalis* (Sasakura et al., 2003), we took advantage of the rapid development of *L. pictu*s to screen for integration. In these experiments, we compared the change in fluorescence across larval development and shortly after metamorphosis between embryos injected with only the reporter construct to embryos injected with the reporter construct and mRNA of the *Minos* transposase (Fig. 1). It has previously been shown that exogenous DNA persists through larval development (Flytzanis et al., 1985) and is largely cleared after metamorphosis. Based on this, we expected differences between transient forms of expression (driven by the plasmid itself) and those driven by bona fide integrations into the genome, would be most pronounced after metamorphosis.

In control larvae injected with only the reporter construct, observable fluorescence declined by ∼10% every 2 days until reaching 3.6% in animals post metamorphosis. In larvae with both the reporter construct and *Minos* transposase, the percentage of larvae with fluorescence also dropped ∼10% every 2 days until 14 days post-fertilization, stabilizing at around 50% (Fig. 1). Consistent with our expectation, the most significant change in the percentage of individuals with fluorescence between experimental (47.4%) and control (3.57%) animals was after metamorphosis: a 43.83% difference (Welch's two-sample *t*-test, *P*=<0.0001, d.f.=5.28, *t*=8.07). Statistically significant differences between experimental groups were also found from days 2 through 8 (Welch's two-sample *t*-test, *P*=<0.05), although the observed qualitative differences between groups (i.e. number of cells expressing the transgene and brightness) was difficult to distinguish until close to metamorphosis (Fig. 1).

**Fig. 2.**
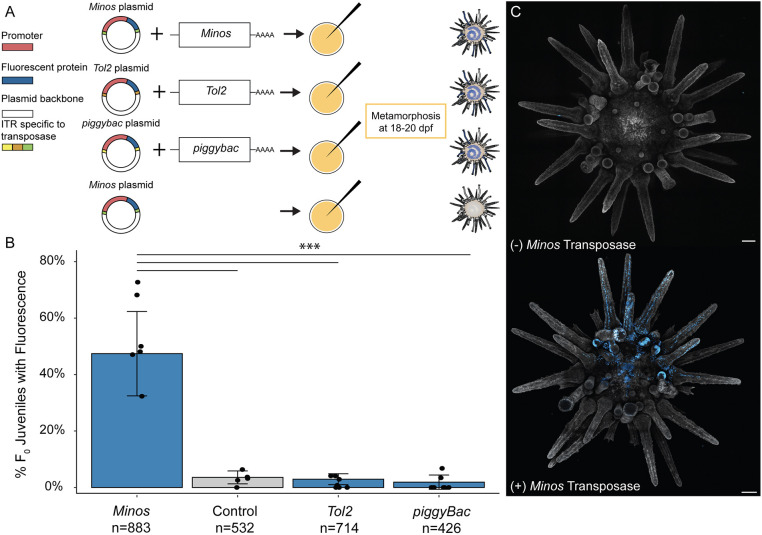
**The *Minos* element resulted in significantly higher somatic integration rates than *Tol2*, *piggyBac* or controls.** *Tol2* and *piggyBac* were not significantly different from controls. (A) Each transposon was tested by injecting mRNA of the transposase along with a plasmid containing a polyubiquitin promoter upstream of a human H2B-mCerulean. Control conditions contained only the plasmid. (B) Screening for somatic integration was performed 2-4 days post-metamorphosis. Each data point represents the proportion of offspring with fluorescence from a unique mate pair. Data are mean±s.d. At least five different mate pairs were used for each experimental group (i.e. different transposon tested). ***P<0.0001 (pairwise Wilcoxon rank sum test, Bonferroni-corrected). (C) F_0_ juveniles injected with the reporter plasmid and with (+) or without (−) the *Minos* transposase. Juveniles were stained with CellMask plasma membrane orange to create contrast with nuclear CFP signal. Scale bar: 100 μm.

Next, we determined whether other transposases might also be effective at introducing similar, stable somatic integrations. *Tol2* and *piggyBac*, which are two commonly used transposable elements used to generate transgenic animals, were tested. We generated LvPolyUb::H2B-CFP constructs for each of these and tested their effectiveness at introducing somatic integrations in juveniles. Neither *Tol2* (3.0%, *n*=714) nor *piggyBac* (1.9%, *n*=426) yielded integration rates significantly different from control conditions (3.6%, *n*=532) (Pairwise Wilcoxon test, *P*=>0.05) (Fig. 2). *Minos* was the only transposase with significantly different somatic integration rates compared with control conditions, *Tol2* and *piggyBac* (Pairwise Wilcoxon test, *P*=<0.05). The efficiency of somatic integration using *Minos* ranged from 32-72% with an average of 47% (*n*=883).

**Fig. 3.**
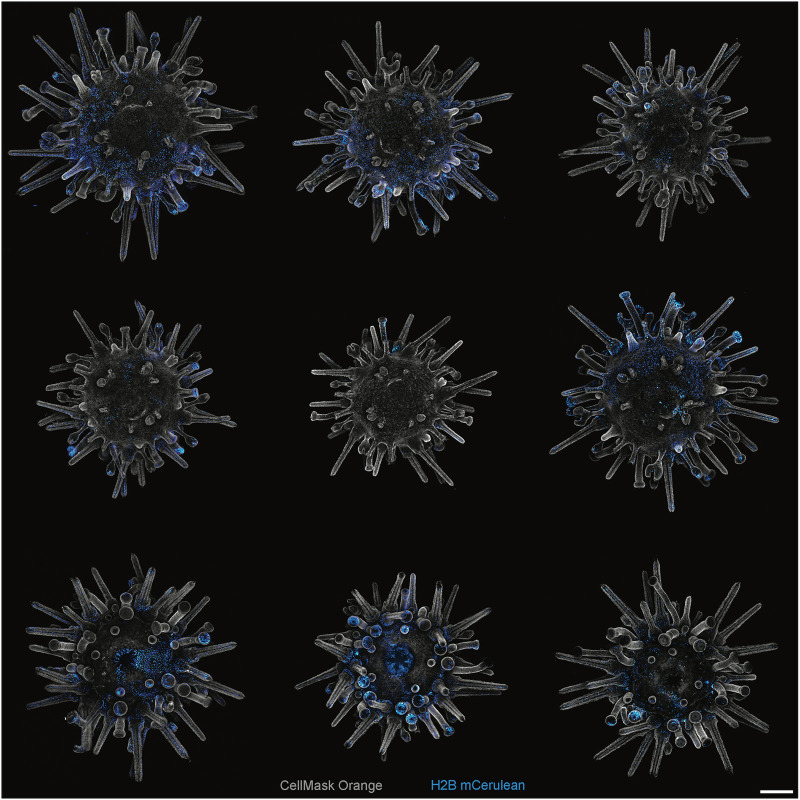
**Mosaicism and variation of transgene expression in F_0_ animals.** Live imaging of nine unique F_0_ juveniles injected with *Minos* transposase and our reporter construct. Top and middle rows are aboral views; bottom row is the oral view. Juveniles were stained with CellMask plasma membrane orange to create contrast with nuclear CFP signal. Scale bar: 250 μm.

Given these results, we used *Minos* to produce transgenic adults to assay for germline integration. F_0_ juveniles exhibited strong, mosaic transgene expression patterns that persisted through to adulthood (Fig. 3). To determine whether the somatic integrations we observed in juveniles might also pass through the germline, we raised 70 fluorescent F_0_ juveniles through to sexual maturation. At approximately 4 months after metamorphosis, we qualitatively graded these individuals based on their fluorescence. Of these, 48 had moderate to strong mosaic transgene expression patterns. These 48 animals were subsequently spawned and crossed with wild-type animals to determine whether their gametes produced transgenic larvae. Of the 48 individuals, we obtained 27 males and 21 females. Interestingly, we observed that sperm from 10 of the males produced transgenic larvae, whereas eggs from only two females produced transgenic larvae. To determine whether this reflected transgene integration, or whether it might be mediated by some form of female-specific transgene silencing, we determined the presence of an integration by PCR. We found that all animals that produced progeny with observable transgene expression were also positive by PCR, and all animals without observable transgene expression were negative by PCR (Fig. S1).

**Fig. 4.**
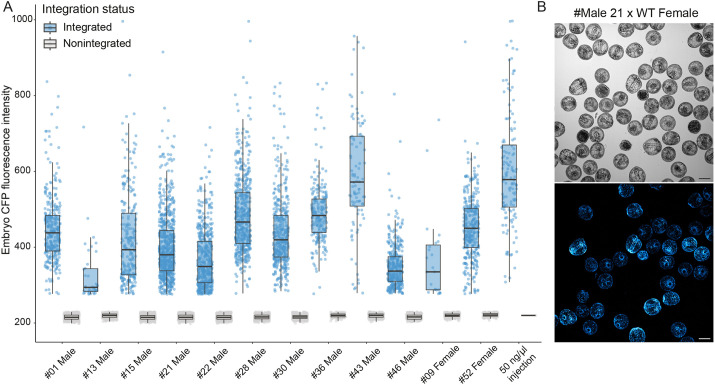
**Measurement of transgene expression levels by high content screening of F_1_ embryos.** (A) Max fluorescent intensity of transgenic embryos separated by parent with germline integration. Each dot represents one embryo at 24-26 h post fertilization. Box plots show median values (middle bars) and first to third interquartile ranges (boxes); whiskers indicate 1.5× the interquartile ranges; dots indicate data points. (B) Representative image used for machine learning-based automated segmentation to determine integration status and measure CFP fluorescent intensity. The top and bottom images are the same embryos in transmitted light and confocal, respectively. Scale bar: 50 μm.

We then sought to determine the level of transgene expression across individuals with germline integrations, in order to determine the timing of transgene expression and characterize transgene expression patterns in F_1_ larvae and juveniles. To determine the percentage of transgenic offspring produced from each individual with germline integration, we used a high-content imaging approach (Tjeerdema et al., 2023). We screened 353-1295 F_1_ embryos from each outcross of the 12 F_0_ animals with germline integration (Fig. 4). The average germline transmission rate was 41.81% (*n*=3826/8579) and varied from 6.26% to 64.30% (Table 1).

**Table 1.**
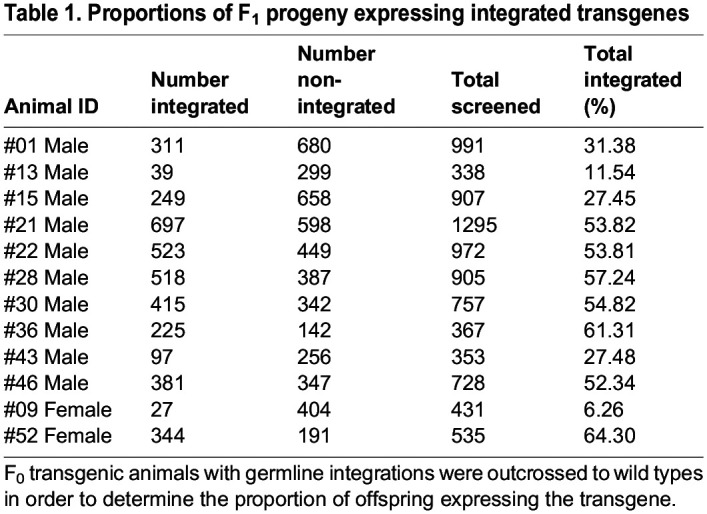
Proportions of F_1_ progeny expressing integrated transgenes

Next, we examined the relative level of transgene expression across individuals and compared transgene expression to overexpression of histone CFP by injection of mRNA, which is the current standard methodology for expression of a histone marker in sea urchins (Fig. 4). All transgenics produced brightly fluorescent embryos that could be easily distinguished from controls by eye, although transgene expression varied 2.5-fold from the brightest to the dimmest individuals. Of these, the brightest transgenic embryos were in the range of a 50 ng/μl mRNA overexpression concentration (Fig. 4) and were more than adequate for visualization in routine confocal microscopy.

**Fig. 5.**
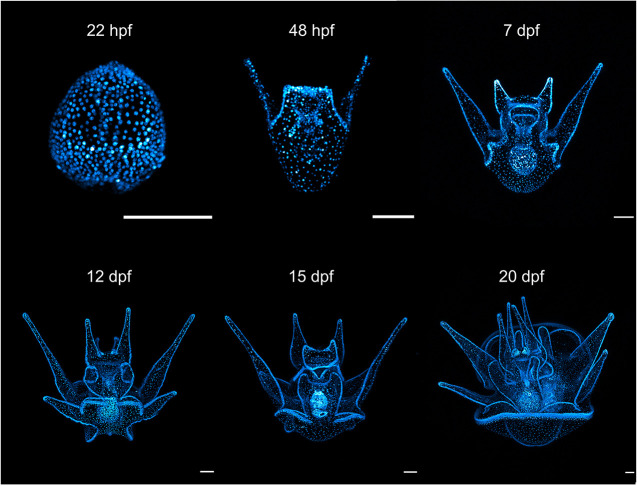
**Transgenes are robustly and ubiquitously expressed in F_1_ embryos and larvae through metamorphic competency.** Image shows confocal micrographs of F_1_ embryo and larvae produced from mating wild-type female eggs with sperm of a transgenic male with germline integration. Scale bars: 100 μm.

**Fig. 6.**
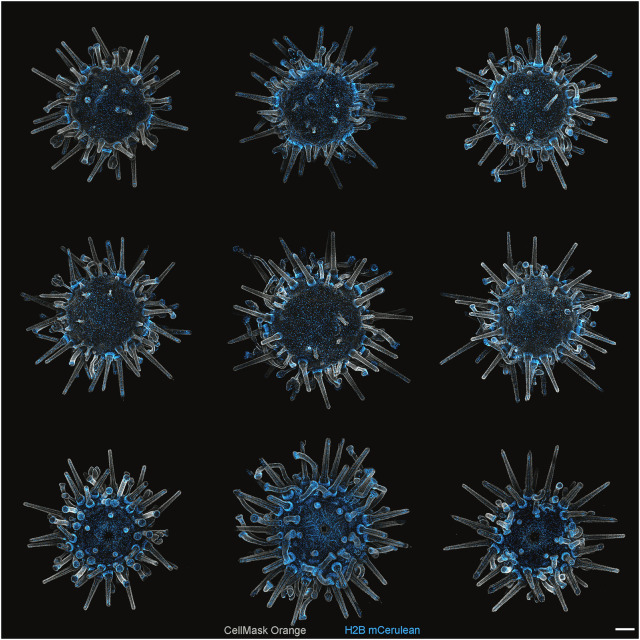
**Transgene expression in F_1_ animals is ubiquitous across the body.** Live imaging of nine unique F_1_ juveniles. Top and middle rows are aboral views; bottom row is the oral view. Juveniles were stained with CellMask plasma membrane orange to create contrast with nuclear CFP signal. Scale bar: 250 μm.

Finally, we examined the timing at which the transgene becomes expressed and whether it persists through metamorphosis in F_1_ progeny. Whether driven through the male or female germline, expression of this construct in F_1_ progeny begins at hatching (∼9 hpf at 20°C) and persists through development (Fig. S2). Nuclei remained brightly labeled through larval development, including in competent larvae that were approaching settlement (Fig. 5). Similarly, transgene expression persisted after settlement and into juveniles of the F_1_ generation, with some variation among individuals (Fig. 6). In many individuals, the expression appeared ubiquitous, at least as gauged by observation on the microscope.

## DISCUSSION

This is the first study to demonstrate stable germline transgenesis in sea urchins, or indeed in any echinoderm. Before this study there was no consensus about how to stably integrate exogenous DNA into the genome of a sea urchin. The first attempt at transgenic manipulation in sea urchins was performed by microinjection of naked DNA (Flytzanis et al., 1985; McMahon et al., 1985). Both circular and linearized plasmid DNA were microinjected into embryos with only a few percent of the injected sea urchins retaining the injected constructs after metamorphosis, and germline integration was not demonstrated. We similarly observed very low levels of fluorescence in juveniles injected with plasmids in the absence of transposases (Fig. 2). However, when using the *Minos* Tc1/*mariner* element, we observed an order of magnitude greater rate of integration compared to injecting the plasmid alone (Fig. 2). This reliably produced high levels of somatic integration in ∼50% of the injected F_0_ juveniles. Somewhat unexpectedly, somatic integration rates using *Tol2* and *piggyBac* were nearly the same as injecting the plasmid alone, suggesting that these transposons are not effective in sea urchins.

One of the biggest obstacles for developing a transposon-based integration method in sea urchins is being able to delineate between expression from the injected plasmid versus expression driven from a stably integrated transgene. Our results indicate that around 12 days post fertilization in *L. pictus*, the difference becomes more apparent (Fig. 1). Two qualitative differences between *Minos*-injected larvae and controls are the level of fluorescence and number of fluorescent cells, both of which appear higher in larvae injected with the *Minos* transposase. We assume that the most likely reason for this is the dilution of the circular plasmid with cellular division, leading to a weaker fluorescent signal as the animal grows (Flytzanis et al., 1985). However, we found screening for integration was ultimately most conclusive in juveniles, because transient plasmid expression nearly disappears at metamorphosis (Fig. 2).

In this study, we noted some possible differences in germline transmission between males and females. The most obvious of these was the disproportionate number of males compared with females with germline integration. The second observation, which may or may not be related to the first, is the difference of transgene variegation of expression (Fig. S3). Specifically, we observed more variegation in the fluorescence levels of F_1_ larvae from females. We speculated that this might be due to some sort of female-specific silencing of the integrated transgene in females. However, our analyses did not support this hypothesis, as all females lacking the transgene expression also lacked integration by PCR. Thus, alternative explanations might be that the female germline is somehow more resistant to integration or that this difference was simply an artifact.

In both males and females, transgene expression was first observed immediately before hatching (Fig. S2), which occurs at ∼8.5-9.5 hpf, depending on temperature (about 9.5 h at 20°C). This corresponds to the major wave of zygotic gene expression in sea urchins (Chassé et al., 2018; Tadros and Lipshitz, 2009). Fluorescence is relatively low at first and is present in only a few cells, but then rapidly increases in intensity and distribution. In many embryos, all cells visible appeared to express the transgene by 12 h after hatching. This absence of expression in eggs and early embryos was somewhat unexpected, as the publicly available RNA-seq data show that polyubiquitin transcripts are present in the egg (Fig. S4) and recruited to polysomes at fertilization (Chassé et al., 2018). One possible explanation for this is the absence of a polyubiquitin-C 3′UTR element in our transgene cassette, as this may include regulatory motifs responsible for maternal deposition as well as stability and translation during early embryogenesis (Gildor et al., 2016). However, for other ubiquitously expressed genes, such as ubiquitin, maternal deposition of eGFP into zebrafish eggs was ultimately observed in the F3 generation using a promoter excluding the 3′UTR element (Mosimann et al., 2011).

Stable transgenesis has the potential to reshape the landscape of sea urchin research. Stable transgenesis offers significant advantages over less-reproducible and repetitive transient methods currently used in the field. For example, this study demonstrates how marker transgenes, such as histone or membrane markers, can be introduced by simply fertilizing wild eggs with transgenic sperm, rather than by overexpression of mRNA. As sea urchins make abundant gametes, and *L. pictus* sperm can easily be cryopreserved or extended (Vacquier and Hamdoun, 2024), virtually any lab will be able to easily make a surplus of transgenic embryos by simply fertilizing eggs with transgenic sperm. In addition, we have shown that the marker transgenes persist into later development, opening the door to live imaging of later larval and rudiment features that have until now only been accessible by fixation (Formery et al., 2023). Perhaps the most significant advantage of all is that transgenesis takes better advantage of the specific biological features of the sea urchin that motivated its use in the first place – i.e. extreme fecundity and developmental synchrony. Both can be more fully used by stable transgenesis through which millions of modified embryos can be generated and used in high-throughput format (Tjeerdema et al., 2023). This is in stark contrast to the current situation where only tens or, at most, hundreds of embryos, are modified and analyzed.

This work is the result of systematic steps to de-risk *L. pictus* as a genetically enabled sea urchin. The outcomes are likely to have significant impact on both the sea urchin and echinoderm community at large, through the production of new animal resources and tools. Importantly this opens the door to the generation of diverse sea urchin lines that can be shared among labs and thereby usher in a new era of collaboration. As such, sea urchin transgenics may dramatically impact the throughput, reproducibility and utility of this iconic animal model.

## MATERIALS AND METHODS

### *Lytechinus pictus* husbandry

Adult *L. pictus* were collected in San Diego, CA, USA and housed in flowing seawater aquaria at 20-22°C. The materials and methods used to spawn, fertilize and culture *L. pictus* were as previously described (Vyas et al., 2022). Juveniles at ∼2 mm size were transferred to a recirculating sea water system (Aquaneering, San Diego, CA, USA) and fed Pacific dulse (*Palmaria palmata*) until they reached ∼1 cm in size, at which time they were fed *Macrocystis pyrifera*. F_0_ adults were spawned by injecting 50 μl of 0.2 μm filtered 0.55 M KCl to collect gametes.

### Promoter selection and testing

Publicly available RNA-seq data on Echinobase (Arshinoff et al., 2022) from *Strongylocentrotus purpuratus* and *Lytechinus variegatus* were used to find candidate genes for promoter discovery. Candidate genes were chosen based on two criteria: (1) transcript per million (TPM) values of at least 200 across all developmental time points and adult tissue types; and (2) previous evidence of ubiquitous expression in other animal models. NCBI BLAST was used to identify the homologous *L. pictus* genes from *S. purpuratus* or *L. variegatus* as the query sequence. Based on these criteria, LOC121415894 (polyubiquitin-C) and LOC121418990 (polyA binding protein) from *L. variegatus* were chosen, and LOC129259176 (tubulin alpha-1A chain), LOC129261990 (elongation factor 1-alpha) and LOC129265555 (polyA binding protein) from *L. pictus* were chosen for promoter screening. The putative promoter region for each gene was chosen to be between 2000-5000 nucleotides surrounding the transcriptional start site, which was synthesized by Twist Bioscience or GENEWIZ from Azenta Life Sciences and subsequently cloned upstream of a H2B-mCerulean (Addgene, 198059). Promoter activity was scored by microinjecting 25 ng/μl of circular plasmid and screening for fluorescence in embryos 24 h post fertilization (hpf). DNA concentrations were determined using a Qubit 4.0 before microinjection.

### Transposon plasmid construction, mRNA synthesis and microinjections

Three plasmids were required to make mRNA of each transposase (*Minos*, *Tol2* and *piggyBac*). The *Minos* transposase (pBlueSKMimRNA) plasmid was a gift from Michalis Averof (Addgene, 102535). The *piggyBac* transposase (pT7mRNA-PB transposase) was purchased from VectorBuilder and the *Tol2* transposase came from the pCS2FA-transposase plasmid (Mackey et al., 2020). Three separate plasmids were designed and synthesized by Twist Bioscience or GENEWIZ from Azenta Life Sciences that were compatible with each transposase. These plasmids contained inverted terminal repeats specific to each transposase and a multiple cloning site. Restriction enzyme cloning was used to clone the promoter of an *L. variegatus* polyubquitin-C gene (LOC121415894; Addgene #218982) upstream of a cyan fluorescent protein (CFP) mCerulean (LvPolyUb::H2B-CFP) inserted into each transposon plasmid (Addgene, #218983) (Fig. S4). All plasmids were sequenced before experimentation through services provided by Plasmidsaurus or Primordium Labs.

*In vitro* transcription using the *Minos* and *piggyBac* transposase plasmids was performed using the mMESSAGE mMACHINE T7 Ultra Transcription Kit (Invitrogen) according to the manufacturer's protocol, and *in vitro* transcription using the *Tol2* transposase plasmid was performed using the mMESSAGE mMACHINE SP6 kit (Invitrogen) following the manufacturer's protocol. mRNA concentration was determined using a Qubit 4.0 and mRNA quality was determined using an Agilent Tapestation RNA Screentape. Only mRNA with RNA integrity numbers (RIN) 8 or greater was used in experiments. Microinjections were performed on one-cell embryos containing a mixture of 500 ng/μl of transposase mRNA, 20 ng/μl of circular plasmid and 1 ng/μl Rhodamine B dextran. Control injections were performed using the same microinjection solution excluding the transposase mRNA.

### Larval and juvenile screening and for somatic and germline integration

F_0_ screening of embryos, larvae and juveniles was performed on a Zeiss LSM 700 equipped with Zeiss PI 10 or 20× objectives and a CFP filter with a X-Cite Series 120 Q for fluorescent illumination. F_0_ screenings of adults before spawning were performed on a Leica M165 FC stereo microscope with CFP filter and 10×/23× objectives with a X-Cite Series 120PC Q for fluorescent illumination. Animals were subjectively graded (A to D) for strength and area coverage of fluorescence. Animals with strong to moderate (A-C grade) observable fluorescence were subsequently spawned and outcrossed with a wild-type male or female to determine germline integration. F_1_ embryos were screened 2 hpf (∼4 cell stage) and ∼24 hpf.

A subset of F_1_ embryos from each outcross were also collected ∼24 hpf for DNA extraction and PCR. DNA was extracted using PureLink Genomic DNA Mini kit (Invitrogen) following the manufacturer's protocol. Primers (F1-GGGAGTTGGGGCAAATAATCC and R1-AAAACCTCCCACACCTCCC) were designed spanning the entire transgene integration cassette (4889 base pairs) using Primer3web (version 4.1.0). New England BioLabs Q5 high-fidelity DNA polymerase was used following the manufacturer's protocol for a 25 μl reaction volume using 5-10 ng of gDNA. Thermal cycling was performed in a Bio-Rad C1000 thermal cycler using the following conditions: initial denaturing at 98°C for 30 s, followed by 35 cycles of denaturing (98°C for 10 s), annealing (66°C for 30 s) and extension (72°C for 2 min), and a final extension at 72°C for 2 min. Annealing temperature was based on NEB Tm Calculator recommendation using a default primer concentration of 500 nM. The resulting amplicon of the PCR was 4889 nucleotides (nt). All PCRs included a positive control and a PCR reagent negative control. A total of 10-15 μl of a PCR product was used for gel visualization.

### High content screening of transgenic larvae

Transgene inheritance frequency in F_1_ progeny was measured using a semi-automated, high content live-imaging approach. F_0_ animals were spawned and outcrossed with wild-type gametes. Embryos were imaged at gastrulation (24-26 hpf) by deciliation with 0.25 M NaCl and plating in a 96-well imaging plate (P96-1.5H-N, Cellvis). Images were taken in transmitted light and CFP fluorescence (445 nm wavelength, 60 μm pinhole, exposure 100 ms and illumination power 500 mW) using the ImageXpress Spinning Disk Confocal HT.ai (Molecular Devices) with a Nikon CFI Plan Apo Lambda 10× air objective. Machine learning-based automated segmentation of embryo *z*-stack max projection images (IN Carta v2.2, Molecular Devices) was used to count embryos and quantify embryo fluorescence as previously described. Embryos were binned as ‘integrated’ or ‘nonintegrated’ based on a maximum fluorescence of greater than or less than 235, and outliers with max fluorescence >1000 were excluded from analysis. To reduce false positives, we removed ‘edge case’ embryos with maximum fluorescence intensity between 230 and 275 from our analysis ensuring we only assessed correctly binned embryos. The rate of germline transmission (n_integrated_/n_total_) was calculated for each F_0_ parent. Representative max projections were edited in Fiji by changing look up tables to cyan hot, adjusting max and min values, and adding a scale bar.

### Confocal image acquisition

Sea urchin larvae were mounted on a glass slide using clay feet for spacing between cover slip and slide. Larvae were mounted live in FSW with approximately 1:4 volumes of 1 M MgCl_2_. Images were taken on a Leica TCS SP8 laser scanning confocal (APO 20×/0.70 CS dry objective) using a HyD detector at a 448 nm wavelength. Imaging parameters were set to a pinhole size of 2 AU, line average 4 and gain 30.0%. For larger larvae, the tilescan function of the Leica Application Suite X 3.5.7.23225 was used, merging together images with a 20% overlap. Juvenile sea urchins (0.5-1 mm) were imaged live in FSW with approximately 1:4 volumes of 1 M MgCl_2_ in a Cellvis 96-well glass bottom plate (#1.5 high performance cover glass 0.17±0.005 mm). Before adding the MgCl_2_, the animals were incubated in 3 ml of FSW containing 0.3 μl of stock concentration (5 mg/ml) CellMask plasma membrane orange (Invitrogen) for 15 min. Images were taken on a ImageXpress Spinning Disk Confocal HT.ai (Nikon CFI Plan Apo Lambda 10× air objective) using CFP (445 nm wavelength, 60 μm pinhole, exposure 100 ms, illumination power 500 mW) and TRITC (555 nm, exposure 50 ms, illumination power 500 mW) filters. Images were edited in Fiji by projecting *z*-stacks with max projection, look up tables changed to cyan hot, max and min values adjusted, and scale bar added (Preibisch et al., 2009; Schindelin et al., 2012).

### Fluorescence screening and statistical analysis

Larval screening was carried out using three or four different mate pairs with 50-100 injected embryos per mate pair totaling 200 embryos for each timepoint (day 2-16) and experimental group (+ *Minos* transposase,− *Minos* transposase). Welch's *t*-test was performed between timepoint (day) and experimental group to determine significant differences. Transposon testing at the juvenile phase was performed using at least five different mate pairs of injected embryos for each experimental group. Each experimental group (*Minos*, *Tol2*, *piggyBac* and controls) had a total of at least 400 juveniles screened for fluorescence. Kruskal–Wallis rank sum test followed by a pairwise Wilcoxon rank sum test was performed to determine statistical significance between groups. Integration efficiency (n_fluorescent_/n_total_) was calculated using a weighted average to account for sample size differences between mate pairs.

## Supplementary Material

10.1242/develop.202991_sup1 Supplementary information
