# Supplementary material for: Stable germline transgenesis using the Minos Tc1/mariner element in the sea urchin Lytechinus pictus
Source: Development. 2024 Aug 19;151(20):dev202991. doi: 10.1242/dev.202991 (PMC11361634; doi:10.1242/dev.202991)
Supplement: Supplementary information [file develop-151-202991-s1.pdf]

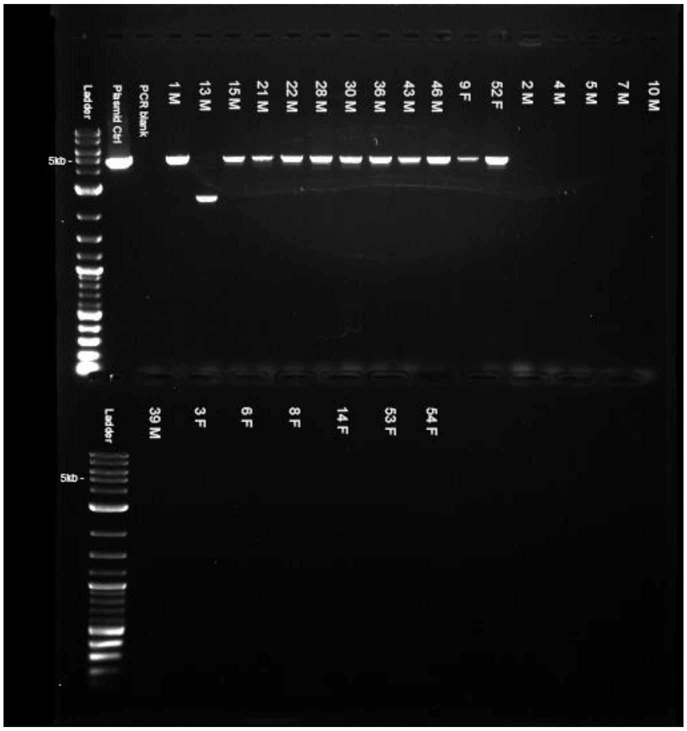

Fig. S1. Representative PCR gel from DNA extracted from F_1 embryos. F_0 adults were spawned and outcrossed with a wildtype male or female to determine germline integration. DNA extracted from F_1 embryos at ~24 hours post fertilization. The number corresponds to the animal ID. M = male and F = female.

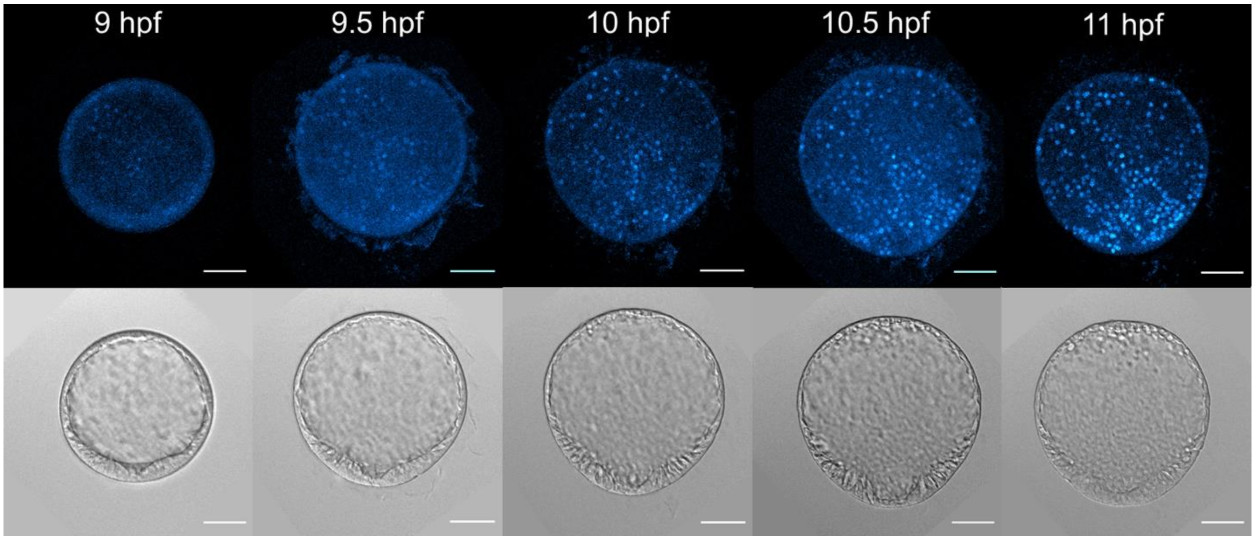

Fig. S2. Transgene expression is visible around hatching. Image shows confocal and transmitted light micrographs of nuclear CFP signal from the same embryo around the period of hatching which occurs ~9.5 hpf at 20°C. Nuclear signal becomes visible in a few cells just prior to hatching (9 hpf). The nuclear signal builds in fluorescent intensity and becomes more visible across the embryo as development proceeds. Scale bars = 50μm.

Development • Supplementary information

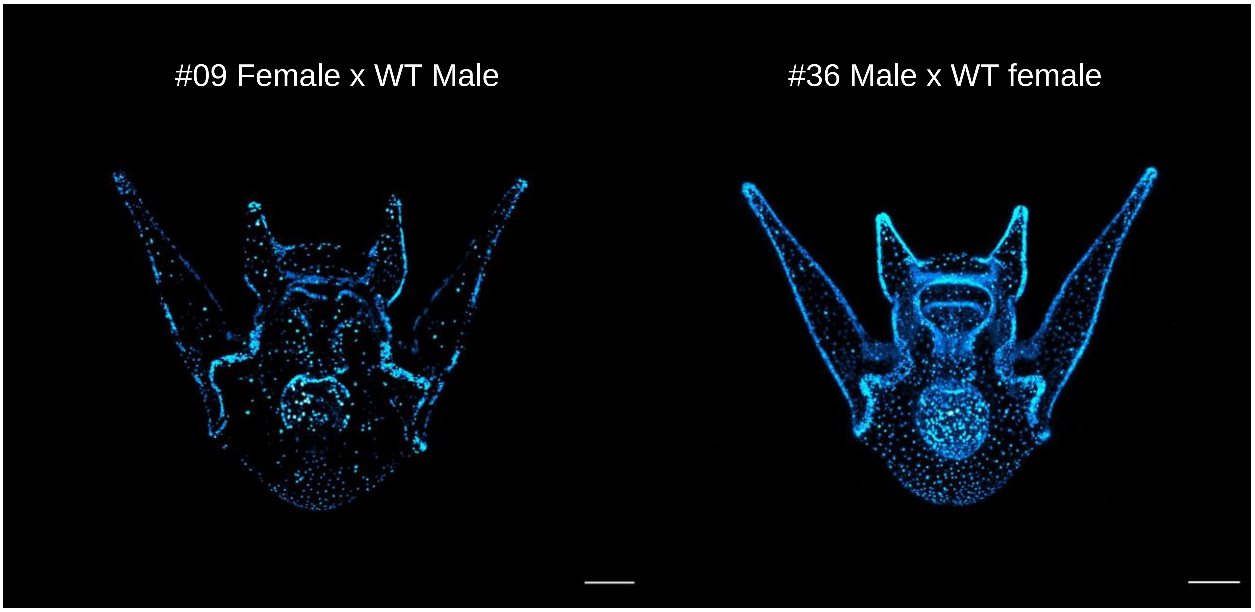

Fig. S3. Difference in variegation of transgene expression in F_1 offspring. The two females with germline integration in this study exhibited greater variegation of transgene expression compared to the males with germline integration. F_0 adults were spawned and outcrossed with a wildtype male or female. Larvae imaged at 7-8 days post fertilization. Scale bars = 100μm.

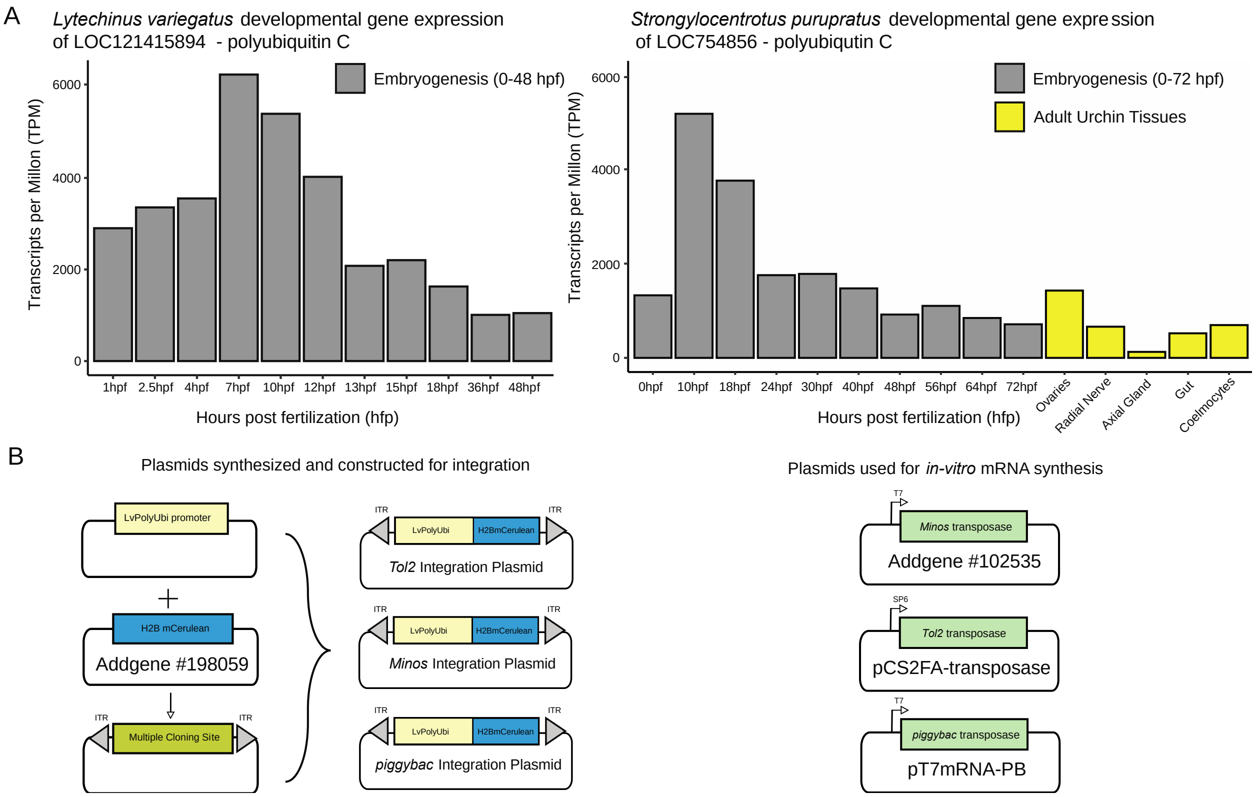

Fig. S4. Promoter discovery and plasmid design for transposon-mediated integration testing. (A) Gene expression pattern of polyubiquitin *L.variegatus* and *S.purpuratus* over early development, (B) Plasmids used and generated for integration.

Development • Supplementary information
